# Why do I have to suffer? Symptom management, views and experiences of persons with a CPTSD: a grounded theory approach

**DOI:** 10.1186/s12888-018-1971-9

**Published:** 2018-12-19

**Authors:** Manuel P. Stadtmann, Andreas Maercker, Jochen Binder, Wilfried Schnepp

**Affiliations:** 1 Department of Health, University of Witten/Herdecke, Alfred-Herrhausen-Straße 50, 58448 Witten, Germany; 2 Department of Psychology, Psychopathology and Clinical Intervention, University of Zurich, Binzmühlestrasse 14/17, 8050 Zürich, Switzerland; 3 Centre for Trauma Disorders, Integrierte Psychiatrie Winterthur, Technikumstrasse 81, Winterthur, Switzerland

**Keywords:** Complex post-traumatic stress disorder, CPTSD, ICD-11, Psychiatry, Symptoms, Mental health, Grounded theory, Experience, View

## Abstract

**Background:**

For the 11th version of the International Classification of Diseases, a new stress related diagnosis has been proposed: complex post-traumatic stress disorder (CPTSD). It is described as a chronic condition with several severe and concurrent symptoms. In the literature, these symptoms are discussed as a common reason for seeking psychiatric treatment as they can influence and impair the quality of life not only for affected persons but also for their social and familial system.

**Aim:**

This research studies symptom management in everyday life by exploring and reconstructing the views, perceptions, experiences, facilitators and barriers of adults with CPTSD.

**Methods:**

A theoretical sampling was used to recruit 18 to 65 years old patients diagnosed with CPTSD from an inpatient setting. The 17 semi-structured interviews were audio recorded and transcribed verbatim. The transcriptions were uploaded into MAXQDA, and a Grounded Theory method based on Corbin and Strauss was used to analyse the data.

**Results:**

We provide a process model with 5 interacting phases: trauma experience, emotional ignorance, overcompensation, paroxysm, and perspectives. Each phase is specified with subcategories.

**Conclusions:**

The participants did not recognise their symptoms as such and were unaware of their diagnosis for many years. Nevertheless, they used various resources and were able to develop skills and techniques to deal with their symptoms and to function on a day-to-day basis. Overall, the process of symptom management was extremely exhausting for the participants and they felt left alone with it. The participants were eager to gain support from healthcare professionals and, when necessary, financial support from the government. Thus, these results indicate an essential need to develop support and tailored interventions for the symptom management of persons with a CPTSD.

**Trial registration:**

Ethical approval was obtained from the Swiss Cantonal Ethic Commission (Nr 201,500,096). This research was also registered at the World Health Organization Clinical Trials Search Portal through the German Clinical Trial Register, Trial DRKS00012268.

## Background

Over the past decades, there have been approaches to integrating the diagnosis of complex posttraumatic stress disorder (CPTSD) as first proposed by Herman [1]. In 2018, the World Health Organization (WHO) is expected to approve the 11th edition of the International Classification of Diseases (ICD-11) as the official latest version [2], and it will contain the new diagnosis of CPTSD.

Research has proposed using core PTSD (post-traumatic stress disorder) criteria to diagnose CPTSD [2–7]. The prerequisite for PTSD is that the affected person develops the symptoms after a stressful situation of exceptional threat or catastrophic extent. This situation can be, for example, abuse of physical or mental nature, a natural catastrophe, accidents or war experience. Further two symptoms from the following three symptom domains must be identified: re-experiencing (RE), avoidance (AV), and sense of threat (TH) [2, 3, 5, 7, 8]. Re-experiencing describes symptoms where the affected person suffers, for instance, from uncontrollable memories of the traumatic event. The domain avoidance describes symptoms where those affected cannot be exposed to situations that could recall the traumatic event. Sense of threat describes symptoms such as perceptions of heightened current threat, hypervigilance, and feeling keyed-up [4, 9–12].

In addition to the above PTSD criteria, a diagnosis of CPTSD requires the presence of symptoms from the following three domains: a negative self-concept (NSC), disturbances in relationships (DR), symptoms of affective dysregulation (AD) [2–7]. A negative self-concept comprises low self-esteem, negative beliefs due to traumatic experiences and feelings of guilt and shame [4, 9, 12, 13]. The same authors described disturbances in relationships as being based on the lack of skills to build and maintain close social relationships. Also, they defined the domain affective dysregulation involving symptoms such as self-harming behaviour, dissociation, emotional numbness, anger outbursts, irritability, excessive crying, or anhedonia. The traumatic event does not necessarily cause immediate distress [2], the symptom onset can be delayed more than six months post trauma [2]. In a previous study, we have provided first results for the symptom burden in psychiatric inpatients with a CPTSD [14]. We could identify several adverse factors, for instance a high prevalence of unemployment, a single status, and living alone with no social support. The results were in adherence with the findings from other studies [4, 12, 15, 16]. Further we have provided results concerning the high educational level of the affected persons, comorbidity in form of several additional diagnoses, multiple trauma experiences, diverse types of trauma experience, and a high level of symptom burden measured by different validated assessment instruments [14].

Symptoms are discussed in the literature as a common reason for seeking treatment, and patients rarely describe one single symptom to their clinicians [17–20]. A symptom is described as a subjective sensation that may vary over time, has antecedents, influences outcomes, and may be influenced by an intervention [20–23]. The same authors describe symptom clusters as having the same characteristics as a symptom. In addition, these clusters consist of three or more concurrent symptoms, are a stable group of symptoms, have a temporal dimension, and are independent of other clusters [21–24]. During the trajectory of illness, affected individuals often experience multiple co-occurring and contradicting symptoms (e.g. flashbacks, hyperarousal, fatigue, sleep disturbance) [19, 21, 24]. These symptoms can strongly influence and alter life quality and daily life itself [24, 25]. Additionally, when symptoms remain underdiagnosed and undertreated, they have a negative impact on patient-reported outcomes including functional performance, cognitive status, and quality of life [20–22].

Recent research shows that handling symptoms and the resulting difficulties are often left to the responsibility of patients and their relatives, and decisions are necessary on how to deal with symptoms, such as when to contact healthcare providers [19, 24, 26, 27]. There is a need for paths to manage symptoms and improve quality of life and overall functioning, especially for individuals and families living with chronic conditions [21–23]. Nonetheless, symptom cluster research is limited, and science is only beginning to understand how to investigate symptom clusters by developing frameworks and new methods and approaches [20, 22]. Further research to identify the mechanisms and processes that underlie symptom clusters is essential in order to develop targeted interventions [21, 23, 28].

For individuals affected by CPTSD, there are two further issues that need to be addressed. There is currently little information on the treatment regimen for patients with CPTSD, and no statement can be made on the differential effect of individual trauma-focused procedures in CPTSD [3–5, 29, 30]. Processing traumatic experiences appears not to be possible without symptom management for the patients [31, 32]. However, no study has been found that dealt with symptom management in the everyday life of adult patients with CPTSD. This study therefore focuses on adult patients with CPTSD and explores and reconstructs their views, perceptions, experiences and the facilitators and barriers as well as the processes involved in symptom management in everyday life. The results can be used to generate more detailed research questions, which could, at best, result in improved patient treatment. These results could also serve as a basis for further research into developing interventions to improve symptom management in everyday life.

## Aims

This study aimed to explore and reconstruct the views, perceptions, experiences, facilitators and barriers of adults with CPTSD in symptom management in everyday life.

## Methods

### Study design

This study is part of larger mixed-method research to investigate symptom management and the social process of adult inpatients with CPTSD [33]. The literature [34, 35] recommends Grounded Theory as a suitable method to investigate phenomena with little scientific evidence. This is the case with CPTSD and symptom management. The Grounded Theory approach was based on the form proposed by Corbin and Strauss [34]. This form claims to outline a more linear and structured approach, in which rules rather than interpretations play a significant role, with the intention to make the analysis understandable, comprehensible and verifiable [35].

### Framework

The study is based on a systemic approach. Chronic illness affects many daily activities [23, 36–39]. It not only affects the person in need but also the surrounding social and familial system [23, 37, 38]. Coping with CPTSD in the social network cannot be adequately understood without being sensitive to the specific kind of relationship between those who are ill and their relatives [23, 29, 37]. Our primary perspective is systemic in that we see the affected individual as part of a system, a member of an interactional group of people who act and react to each other. We also subscribe to a central position of symptom management research that places the experience with the illness and the individual coping strategies at the very centre of attention [19, 23] and addresses the affected person as an expert.

### Setting

This study was conducted at the psychiatric institution Integrierte Psychiatrie Winterthur, Zürcher Unterland (IPW). IPW is a large, non-profit, community-based organization that provides psychiatric services in the city of Winterthur in the canton of Zurich, Switzerland. It provides a full continuum of clinical and community-based mental health services for individuals with several mental health issues. The current study was conducted at a specialized inpatient mental health ward for psycho-traumatology. The ward treats approximately 200 patients per year. It provides treatment for a diverse adult population from the German-speaking region of Switzerland. The ward has a capacity for 17 patients and has a 24-h shift organization for nurses.

### Sampling and recruitment

Over a six-month period, the participants were chosen from a larger sample of adult inpatients (*n* = 133) who had participated in a previous quantitative study [14]. The Zürcher Cantonal Ethics Review Board approved the study. In order to provide a description of CPTSD sufferers with regard to their symptom management, we aimed at a heterogenous and diverse group of participants using theoretical sampling, with the aim of enabling a rich illustration of relevant aspects of the phenomenon. Inclusion criteria comprised: It was the participants’ first inpatient treatment on the psycho-traumatology ward. They had to be between 18 and 65 years of age. A CPTSD based on the International Trauma Questionnaire (ITQ) must have been diagnosed. A good knowledge of German was required. Also, a relative must have been willing to participate. Relatives were defined as individuals who were self-defined relatives, who may or may not have been bound by blood ties, law, friendship or declared commitment and who shared deep personal connections to the participant and provided various forms of support [40, 41]. Exclusion criteria included acute or latent suicidality of the patient and a main diagnosis other than CPTSD. Patients who might endanger themselves or others were also excluded. Written informed consent was given prior to the interviews. Informed consent was obtained from patients as well as from relatives. In this study we report exclusively patient participants’ experiences and views. The names contained in this manuscript are pseudonyms and have been changed to ensure the anonymity of study participants. The pseudonyms were introduced after the participants were audio recorded and are solely known to the first author of the study.

### Data collection and analyses

The first author, with experience in treating and caring for patients with PTSD, was responsible for the qualitative collection of data with semi-structured interviews. First questions were developed based on the results of the first quantitative phase relating to the level of symptom burden [14]. For instance, asking the sufferers with different levels of symptom burden how they perceived their condition in daily life, or which symptoms they perceived the most impairing or the most difficult. Similarly the domains of the symptom management model [24] were used to develop further questions. For example, what did they do to improve their symptom burden? When did they initiate their strategies? Who delivered support and help? Further questions were also posed, such as how they perceived their quality of life, or how the sufferers perceived their functional status in daily life. The clinician’s internal code allowed the identification of patients, after they were asked for an interview.

To create the largest possible contrast between the interview partners, the participants were chosen using theoretical sampling [42]. After the descriptive analyses and results of the first quantitative study (*n* = 133), the first three participants were specifically identified based on their numeric level of symptom burden (low, middle and high) [14]. Further selection and identification of participants was based on the questions arising during the process and performed during the analyses. For instance, in a second step we identified participants with different trauma experiences. In a third step, we identified participants with no diploma or apprenticeship and contrasted the results in a fourth step selecting participants, who were still employed. The descriptive results of the quantitative study allowed us to identify and choose participants with the characteristics of interest.

The interviews took place after the inpatient treatment. The data collection took place between 1st March 2017 and 31st August 2017 within the psychiatric institution IPW in Winterthur, Switzerland. The semi-structured interviews lasted between 45 and 85 min; an additional 30 min after the interviews were available for questions and explanations. If any psychological crises had arisen due to the interviews, these could have been intercepted by a mental health nurse on the ward. The shift organization on the ward is guaranteed 24 h a day by the nursing team.

The interviews were audio recorded and subsequently transcribed verbatim. To comply with data protection, all names were anonymized. The semi-structured interviews were analysed based on grounded theory as proposed by Corbin and Strauss [34]. In a first step we conducted a line-by-line coding, within a group of Ph.D. students. Second, after using analytical techniques in open coding, the set of categories were reduced and clustered during an axial coding phase. The third coding level consisted of selecting and integrating the categories into a final theory [34, 35, 43]. MAXQDA 12 software was used for this process. To ensure the quality of data analysis, the process itself and the results were regularly discussed in a peer group of Ph.D. students led by an experienced qualitative researcher. Based on the discussions and constant comparative analysis process within this research group, data saturation was achieved after the 15th interview when no new data emerged, and all concepts of the theory were well developed. To ensure no new data emerged, we conducted two further interviews, resulting in a total of 17 interviews. This procedure adheres to current literature, which states approximately 15 participants are needed for data saturation to be achieved [44–46]. We also conducted a member check of our final model with the participants to determine the accuracy of the reported data. All patient participants confirmed having been or still being in one of those phases. The data analysis was undertaken in German, the language in which the interviews were conducted. The final report of the findings was written in English.

## Results

**Fig. 1:**
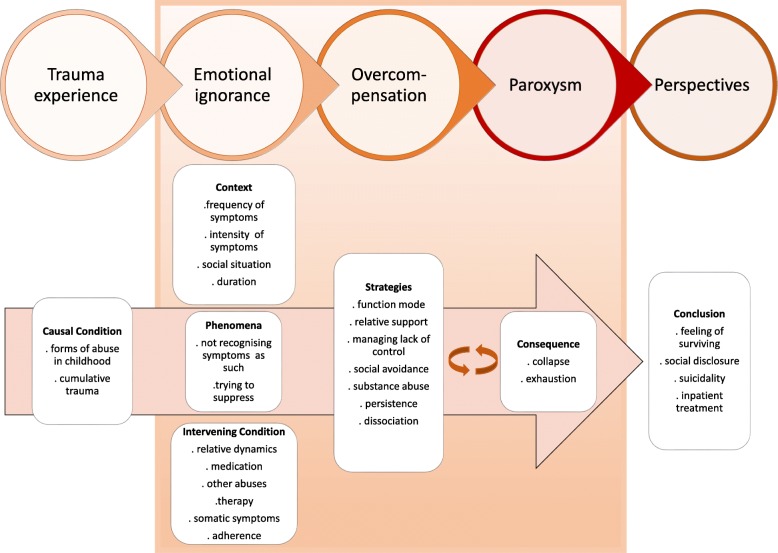
Process model for symptom management in persons with CPTSD

Data saturation occurred after the 15th interview. To ensure no new categories arose from the data, we conducted two additional interviews. We aimed to select a diverse sample to possibly include a multitude of relevant views and experiences and to strengthen the credibility of the findings. Hence our final sample of 17 participants showed heterogeneous sociodemographic characteristics and different levels of symptom burden (Table 1). The data analysis resulted in a process with five categories or themes that were sorted into an explanatory framework that sequenced the progression and experiences of the participants through their life of coping with symptoms of CPTSD. This framework (Fig. 1) is separated into five phases: 1.) Trauma experience, 2.) Emotional ignorance, 3). Overcompensation, 4.) Paroxysm and 5.) Perspectives. These sections describe each of the characteristic categories and subcategories. We additionally provide participant quotes as examples.

**Table 1:** Participants (pseudonyms)

NAME	Gender	Age	Occupation	Trauma experience
Mary	f	40	former nurse	1, 2, 3, 6
Celia	f	60	former teacher	1, 2, 3, 6
Paula	f	22	former teacher	1, 2, 3, 6
Max	m	34	former military	1, 2, 3
Susanne	f	58	former nurse	1, 2, 6
Peter	m	30	economics student	1, 2, 5
Marta	f	48	former smith	1, 2, 6
Sonja	f	48	former airport employee	1, 3, 4
Paul	m	46	no diploma or apprenticeship	1, 2, 6
Yuki	f	47	no diploma or apprenticeship	1, 2, 4
Laura	f	38	bank employee	1, 2, 3, 4
Tim	m	58	former manager	1,3,5,6
Clara	f	19	retail sales person	1,2,3
Laura	f	33	former hairdresser	1,3
Nicole	f	45	self employed	1,2,4
Astrid	f	26	student natural science	1,3,4
Jana	f	62	former branch manager	1,2

Legend: Early Childhood Trauma = 1, Sexual abuse = 2, Physical abuse = 3, Domestic violence = 4, School Violence = 5, Neglect = 6

### Trauma experience

This phase describes the **Causal Condition**. A more specific description is in the sub-categories of different forms of abuse in childhood and the participants’ cumulative trauma experience. All the participants had a story of early childhood trauma and at least two of the following types of traumatic experience: sexual abuse, physical abuse, domestic violence, school violence, neglect, or war.

*“I got the information about soldiers’ illness from the American media… But I didn’t know that it can also develop after physical or sexual abuse” Max, 34*

*“It started in my childhood and then many terrible things happened in my life. I don’t know why I still have to suffer.” Paul, 46*

### Emotional ignorance

This phase describes the main **Phenomena,** and the participants’ experience of not recognising their symptoms as such. Participants identified their symptoms as part of their personality and their being: something natural and given, based on the circumstances they mostly grew up with. They sometimes thought their very self was not strong enough, weak, or just odd compared to other humans. In addition to unawareness of symptoms, the participants also struggled with an inability to regulate themselves and to cope with their condition.

*“I thought it was my personality and I was just weak and useless. Now I know my symptoms were the cause of so many reactions” Sonja, 48*

*“It's like being a ping pong ball blown by a hair dryer. It does with me what it wants; it shoots me to the right, to the left or up or down and I can’t do anything to stop it.” Mary, 40*

Further, the results elaborate the struggle of those trying to suppress and control the unknown and, for them, latent dangerous feelings.

*“Sometimes it was so intense, I was really desperate. I knew there was something else … Something that makes me react differently from others. I do my best to ignore it.” Celia, 60*

The **Context** identifies critical issues such as frequency of symptoms, intensity of symptoms, duration, and social situation. Participants retrospectively experienced a high frequency of symptoms, e.g. every day since their childhood, while the intensity varies. For instance, dissociation had a high impact and intensity in their daily life, while their feeling of worthlessness was described as latent and an almost omnipresent symptom, which eventually was identified as part of their very self. Another important category was the social situation. Participants with an income described a lower level of uncertainty and stress regarding their future. They could also afford to spend money on activities, e.g. movies or inviting their children for lunch in a restaurant, which distracted them, improved their social interactions and helped to release the inner tension. Contrastingly, participants with no secure income or financial support described the feeling of not being a part of society. This situation was described as a context factor that made it difficult for them to cope with their daily life, to manage bills, to manage their administrative tasks, and as a factor that increased symptoms such as rumination and inner tension.

*“ I’ve had symptoms almost every day since childhood. In school, the others thought I was mad, and I must admit I thought so too. Four years ago, I finally realized that I’m not crazy.” Max, 34*

The **Intervening Conditions** describe facilitators and barriers of the patients for symptom management in everyday life, for instance, supportive and caring relatives, with participants describing in diverse ways how relatives play a key role in their life. Individuals related by blood or not were described as a life anchor. Highly important was being able to learn to trust again. Participants who shared some of their traumatic experience with their relatives described talking for the first time: they felt ashamed, unsure, and feared their relatives’ reactions. Experiencing comprehension and compassion helped them afterwards to trust a person and improved their level of symptom burden.

*“But I think it’s nice to have such a good friend. It makes it easier… Just to know there is someone around makes me feel less lonely.” Paula 22*

Otherwise, not sharing the cause of their condition with their relatives also generated feelings of shame and guilt.

*“I can’t… I just can’t. I am too ashamed to tell him (husband). He doesn’t understand many things; I think he might if I told him. Then, I feel guilty about our situation.” Celia 60*

For the participants, support in activities of daily life through their relatives was of great importance. They learned to accept their lower level of performance and to realize they were not alone. Further, during that time, participants could re-vitalize.

*“I couldn’t even get up and do the dishes, but he was there. He also helped a lot in raising the children. He really is a good husband. I wouldn’t be here without him” Marta, 48*

For some, medication was an important facilitator. With the help of different substances, the participants were able to manage their daily life. Where medication was not prescribed, some of the participants tried unknowingly to suppress their symptoms and emotions with substance abuse such as with alcohol, cocaine, or cannabis. This reaction resulted in a barrier, making it more difficult for them to understand and handle their situation. Experiencing multiple abuses during their life was described as a major barrier, due to not being able to give a meaning to the pain and suffering provoked by the traumatic experience.

*“Not only in my childhood, even afterwards many things happened. I always asked myself, why me? I still don’t know why me. Vodka and my joint often helped me to forget.” Clara, 19*

Being able to make use of the right therapy and avoid unnecessary medical interventions was a major facilitator. Then again, being treated for another condition, for instance depression or schizophrenia, instead of for PTSD was a major barrier.

*“I’m angry and sad. I took medication because they thought I had schizophrenia. For many years. Mmm I guess 16 years later I know I have a PTSD and that fits.” Yuki, 47*

Several participants described somatic symptoms such as back pain, headache, stomach ache as being a barrier, which also meant it was not easy for medical professionals to handle and identify the symptoms as possible consequences of trauma.

*“The body sensations are awful. Suddenly my hands are sweaty, or the soles of my feet are wet, bah! The tension in my back is also a nuisance. These body things make it more difficult to deal with” Sonja, 48*

Adherence to diagnosis was also described as a major facilitator in diverse ways. For example, being able to identify the diagnosis and accept it improved the feeling of having a kind of control over the disorder and decreased the feeling of stigma.

*“I now accept the diagnosis, I no longer feel ashamed, because it was not my fault.” Jana, 62*

### Overcompensation

In this phase, the category **Strategies** illustrates participants’ efforts to cope subconsciously with their symptoms, or to compensate for them, for instance with dissociation or persistence.

*“I didn’t know what it was at the time… that tension. I was just not here… often… I didn’t feel my body at all” Mary, 40*

An important sub-category was described as function mode. Mostly participants had a very high level of functioning. They had mostly a higher level of education and, while working, performed efficiently. The reasons for that mode were as follows: The legitimation to be alive based on their school or work performance, the justification as a member of society based on their performance, not having any uncomfortable feelings based on the overcompensation through their workload and seeking confirmation from their professional environment through their performance and qualifications.

*“Distraction I think, and later at 18 sort of functioning. At 16 I started to work in a hospital. Shift work was a great distraction. For many years I had just two days a month off. Yeah…” Susanne, 58*

Other categories such as “caring relatives” were mostly considered important. Sufferers described that having a helpful and supportive relative ameliorated their symptom burden. Hereby different subcategories could be identified, for instance, managing the activities of daily living, such as cooking, doing the dishes or the laundry, shopping, administrative issues, or support with occurring symptoms, such as providing help after being instructed how to deal with dissociation. Another subcategory was emotional support, for example being there for the affected person when needed, being available when the affected person needed someone to talk to. This subcategory also included not questioning whether the experiences of the affected person were real or not, motivating the affected person to keep in contact with others by taking them out for dinner, a walk, or on vacation.

*“Don’t give up and again and again two or three days without sleep and then work again. My wife had to take over many things. That was helpful” Peter, 30*

Managing the lack of control characterized further participant strategies, mostly based on feelings of anxiety and fear.

*“I just scratched myself to the bone, just to calm me down. My parents… They thought it was a skin disease, they thought I had chronic eczema.” Marta, 48*

When the described strategies no longer functioned, the affected people often struggled with social avoidance and substance abuse to reduce their psychological tension. Being mostly at home for years, just going out when necessary and having no social interactions was one method of feeling in control over their situation.

*“But yeah, but yeah somehow, I had nothing, so I went through addiction, I started with alcohol and other drugs. I also preferred to be alone, to make the whole thing better.” Sonja, 48*

### Paroxysm

This phase indicates the possible **Consequence** when overcompensation is no longer working. Paroxysm is described as an uncontrollable outburst, sudden increase, or recurrence of symptoms [47]. That definition complies with the condition described by people affected by CPTSD. In the subcategory collapse, the symptoms can no longer be suppressed and those affected undergo impressions of collapsing and feeling exhausted after strategies used no longer work. Also, feelings of being worthless and of shame and guilt at not being able to perform as they could previously were described. In the subcategory feeling exhausted, the participants described how using their strategies for years consumed most of their energy. Further there was mostly a trigger situation, for example losing their job, a divorce, or an accident. After the event, they felt exhausted and without energy and therefore completely defenceless against and overwhelmed by their symptoms. Additionally, they could not explain to themselves why, apparently, a single event caused such immense reactions and drain of energy.

*“I was totally incapable of working and couldn’t manage my life” Peter, 30*

*“For a long time, I did not think I was ill. For me it was as though I could still do everything. Until I noticed nothing worked anymore, because it’s all so exhausting.” Paula, 22*

### Perspectives

This phase specifies the **Conclusion** of the process. For instance, the subcategory a feeling of surviving has been described as a possible outcome. Participants experienced the development of perspectives, in which they felt they had new goals, a better understanding of their condition, feelings of control, and they had hope again.

*“Because yeaah it’s difficult to describe. In a way it’s liberating. Because yeah, I now know where the problems lie, and I can work on them.” Yuki, 47*

The subcategory social disclosure is a possible result of experiencing different recurrent situations of collapsing and not being able to perform as before. The feeling of disappointment in themselves was also described in this subcategory. Furthermore, feelings of shame and disclosure based on not being able to handle social interactions could be identified. If the affected person was aware of the possibility and had access to a health care provider, a possible result was also inpatient treatment. If there was a lack of support either from the social environment or the health care system, those affected also developed suicidal thoughts.

*“From the age of 22 to 33 it just didn’t exist. It worked unconsciously through nightmares, I suspect. When I was 33 it broke out again and I had a nervous break-down and nearly killed myself.” Susanne, 58*

## Discussion

This is the first known study that highlights symptom management of those affected by CPTSD. Using the data collected through interviews, we developed a conceptual model (Fig. 1) that identifies five major phases experienced by affected individuals: trauma experience, emotional ignorance, overcompensation, paroxysm and perspectives. Those affected described a situation in which they felt something was different in comparison to others. They mostly interpreted this condition as a deficiency in their personality. Therefore, the phenomenon of not recognising their symptoms as such and trying to suppress these as well as emotions is an important result.

Persons suffering from CPTSD also tried to function over a prolonged period. They achieved, sometimes over decades, an elevated level of performance. For instance, at their workplace they focused on doing an excellent job to satisfy customers and superiors. Nevertheless, there were capacity limits. When these were exceeded, support was an urgently needed. The participants in this study criticised a lack of support from the health care system. Some of the participants were either treated based on another diagnosis or they were not treated at all. These findings raise questions about CPTSD awareness in the public health sector.

The individuals considered it extremely important to organize their daily life (e.g. cooking, doing the dishes, cleaning the apartment, administrative issues). Literature also describes relatives as an important and supportive part in the lives of patients with a chronic condition [27, 39, 48]. Our results are consistent with that evidence. On the one hand, support from relatives in the daily life of those affected by CPTSD was described as a major facilitator, for instance, in managing housework, managing administrative tasks and settling payments. Further, emotional support was a vital element during their process of symptom management. It was an important experience for the participants to be able to trust a person again and to realise that they were not alone, to feel respected as a person even though they could not perform as before. On the other hand, relatives could also function as a barrier, for instance, when the affected person felt guilt and shame for not reporting the causes of their illness or when they recognized that their condition caused elevated stress level in their relatives. Another example was when the relatives were overprotective and those affected avoided speaking out to prevent distress. This raises the question of whether specialized educational and support services could be a possible effective intervention for both parties and whether greater family involvement is required. Recent literature suggests this for patients with PTSD [49–51]. Our results indicate that it is also needed for patients with CPTSD.

An important intervening factor was social support in the form of financial support while being unable to work. Those affected by no financial support either from relatives or the welfare state e.g. a disability pension, experienced considerably more difficulties in handling their daily life, and they did not feel part of society. The financial worries often impeded the participants in overcoming their anxiety. This result highlights the importance of support in this field. This result is also consistent with the findings of another study describing a correlation between high rates of unemployment and CPTSD [4, 14].

Retrospectively, some participants described having experienced various phases of collapse through their lives, as illustrated in our model (Fig. 1). After experiencing feelings of exhaustion, difficulties with rising emotions (such as feelings of loss, worthless and shame), and memories, the participants returned to strategies they knew would work. Those strategies gave them the feeling of being in control of their lives. These results are consistent with recent evidence from Karatzias and colleges [52], reporting negative trauma-related cognitions about the self as a most important factor in CPTSD. Likewise, retrospectively, the participants felt regret and anger for not being able to address the real issues causing their condition. Knowledge about their condition gave them feelings of hope and new perspectives. Understanding interactions and reactions to their symptoms gave them a sense of security. Thus, these results indicate an essential need to develop, support and tailor interventions for symptom management of persons suffering from a CPTSD.

This trial also has limitations that should be considered in evaluating the results. The first author designed the questions; it can be assumed he is not value-free and that he inadvertently influenced the results due to his own personal and professional beliefs. These values may influence the conduction and reporting of the research. Other researchers may have generated different results. The nature of qualitative research implies there is no possibility to generalize the results. Based on our study design, our sampling was selective and targeted, focused on participants with different socio-demographics, distinct levels of symptom burden from an inpatient setting and identified during the ongoing analyses. Additionally, contextual influences were not considered in this research. For instance, we do not know if our results apply to other settings in our health care system, to other countries or to other cultures. We realize that the conceptual model does not represent the unique symptom experiences of those with CPTSD. We do not claim causal inferences based on our data and framework. Further, quantitative research required to test our results to determine possible cause and effect or correlations. Research is also needed to expand the conceptual model and to discover attributes specific to each phase. Moreover, the diagnosis of CPTSD was based on self-report with the ITQ. The instrument we applied was the German test version 1.4 which was still under development. Therefore, diagnosis criteria may differ from the conditions for CPTSD as reformulated by the WHO in the new ICD-11. Despite these limitations, our study contributes first results with regard to symptom management of adults with CPTSD and contributes to the growing literature related to CPTSD.

## Conclusion

This study focused on the unique experiences of symptom management of participants with CPTSD. They did not recognise their symptoms as such and did not know their diagnosis for many years. Nevertheless, participants used various resources and were able to develop skills and techniques to deal with their symptoms and to function on a day-to-day basis. Overall, the process of symptom management was extremely exhausting for the participants and they felt left alone with it. The participants were eager to gain support from healthcare professionals and, when necessary, financial support from the government. The results serve to gain a better understanding of the condition. Further, these results could be focal points to developing and researching new interventions, thus improving symptom management and quality of life.

## Abbreviations

CPTSD: Complex post-traumatic stress disorder
DGPPN: German Association for Psychiatry, Psychotherapy and Psychosomatics
DRKS: German Clinical Trial Register
Fig: Figure
ICD-11: International Classification of Disease, 11th version
IPW: Integrierte Psychiatrie Winterthur, Zürcher Unterland
ITQ: International Trauma Questionnaire
PTSD: Posttraumatic stress disorder
WHO: World Health Organization
